# Action dynamics reveal two types of cognitive flexibility in a homonym relatedness judgment task

**DOI:** 10.3389/fpsyg.2015.01244

**Published:** 2015-08-28

**Authors:** Maja Dshemuchadse, Tobias Grage, Stefan Scherbaum

**Affiliations:** Department of Psychology, Technische Universität DresdenDresden, Germany

**Keywords:** cognitive flexibility, priming, homonym, dynamics, continuous time, mouse movements, executive functions, relatedness judgment

## Abstract

Cognitive flexibility is a central component of executive functions that allow us to behave meaningful in an ever changing environment. Here, we support a distinction between two different types of cognitive flexibility, *shifting flexibility* and *spreading flexibility*, based on independent underlying mechanisms commonly subsumed under the ability to shift cognitive sets. We use a homonym relatedness judgment task and combine it with mouse tracking to show that these two types of cognitive flexibility follow independent temporal patterns in their influence on participants' mouse movements during relatedness judgments. Our results are in concordance with the predictions of a neural field based framework that assumes the independence of the two types of flexibility. We propose that future studies about cognitive flexibility in the area of executive functions should take independent types into account, especially when studying moderators of cognitive flexibility.

## Introduction

As humans, we are able to adapt our goals and our behavior to a constantly changing environment. The flexibility to shift cognitive sets is a central part of executive functions (e.g., Miyake et al., 2000; Diamond, 2013). It requires either identifying alternatives related to the original set or overcoming perseveration on previous sets by shifting to a new alternative set. In the research on executive functions, cognitive flexibility is usually studied in two ways. The first approach focusses on task-switching (e.g., Monsell, 2003), set-shifting (e.g., Dreisbach and Goschke, 2004) or related paradigms that reveal switch costs (higher response times) in switch trials—where one has to switch from one task to another one—compared to repeat trials—where the same task is performed again. Several influences on this type of cognitive flexibility have been identified. For instance, it has been found that positive mood increases cognitive flexibility and thereby reduces switch costs (Dreisbach and Goschke, 2004). Interestingly, this influence of positive mood on cognitive flexibility builds the link to the second approach that focusses on the breadth of attention, as studied, e.g., with the flanker task (Eriksen and Eriksen, 1974) or with semantic tasks as the remote associates task (Mednick et al., 1964). The flanker task—in which one has to respond to a central target while ignoring flanking stimuli—shows the congruency effect, so that incongruent trials—in which center and flanking stimuli point to different responses—are slower than congruent trials—in which center and flanking stimuli point to the same response. This congruency effect increases with decreasing distance between center and flanking stimuli (Eriksen and Eriksen, 1974). Interestingly, positive mood also increases the congruency effect similar to effects of positive mood on the detection of remote associates in the remote associates task (Rowe et al., 2007). These findings are interpreted as an increase in the breadth of attention and hence increased cognitive flexibility. The link between the two approaches—shifting and attentional breadth—as indicated by the effects of positive mood is also innate in other conceptualizations of executive functions, i.e., the shielding-shifting dilemma that assumes the shifting of goals and the shielding from distraction as opposing constraints of the cognitive system that span the dimension of cognitive flexibility (Goschke and Bolte, 2014).

Here, we argue, that despite the common influence of positive mood and the apparent similarities of these conceptualizations, the two approaches tackle different types of cognitive flexibility based on independent underlying mechanisms: The ability to identify related alternatives or to completely shift to new alternatives comprise two distinct and independent aspects of cognitive flexibility, that become evident when referring to neural mechanisms. We will call the first type *shifting flexibility*—the ability to overcome perseveration by switching from an active neural pattern representing a certain cognitive set to a new one; and we will call the second type *spreading flexibility*—the ability to identify related alternative cognitive sets based on the spread of activation to nearby neural patterns. A classic example for these two abilities stems from the field of problem solving: Duncker's candle task (Duncker, 1945). Participants are given a box of tacks, matches, and a candle. Then they are instructed to attach the candle to a wall of corkboard and to light it (compare Isen et al., 1987). The “correct” solution is to use the tacks' box as platform for the candle and to attach this platform to the wall using the tacks. However, a typical phenomenon is *functional fixedness*: participants are not able to leave the functional cognitive set associated with the single items: they only explore different functions in the vicinity of the original functions (for example, using the melted wax of the candle as glue). Within this candle task, *spreading flexibility* describes the exploration of all ideas related to the original function of the items, while *shifting flexibility* would mean to shift to a completely new function—leaving functional fixedness.

Decomposing these two types of cognitive flexibility and their underlying cognitive processes experimentally, however, poses a methodological challenge: Typical approaches either analyze inter-individual variance in different tasks (e.g., Miyake et al., 2000) or analyze patterns of lesions or neural activation between different brain regions (e.g., Koechlin et al., 2003; Stuss and Alexander, 2007). Both approaches require a large number of participants and inter-individual variability to differentiate components, and both approaches are correlational in using given inter-personal variation. For example, in their seminal study on components of executive functions, Miyake and colleagues measured 137 participants—all undergraduate students—on 14 tasks, identifying 3 specialized and 1 general components of executive functions via factorial analysis (Miyake et al., 2000). However, in later studies, measuring participants between 20 and 81 years, an additional fourth specialized component was identified (Fisk and Sharp, 2004). While this does not question the original three components, it illustrates that variance between participants is necessary in this approach to identify specific components.

To go beyond such correlative analyses, experimental approaches manipulate task-requirements of different components and measure the resulting differences in response times and error rates (e.g., Braver, 2012). We will embrace and add to this experimental approach by adopting a dynamic continuous perspective (Spivey and Dale, 2004, 2006; Spencer et al., 2009). We manipulate the requirement for the two types of flexibility, shifting and spreading, within one task and compare their effects continuously in time by using mouse tracking (Spivey et al., 2005; Freeman et al., 2008; Scherbaum et al., 2010; Dshemuchadse et al., 2012) which will enable us to distinguish both types by their temporal profiles (Scherbaum et al., 2010). To manipulate and measure the two types within the same task, we will use a relatedness judgment task (Zwaan and Yaxley, 2003). In this task, participants have to detect whether two words are semantically related or not. Hence, participants have to search within their semantic network to detect associations between words (Faust and Lavidor, 2003). For this search process, one can manipulate the level of required spreading flexibility necessary to solve the task by varying the strength of relatedness between words. To also manipulate and assess shifting flexibility, we extended this task by using homonyms (Dshemuchadse, 2009)—words with two or more different meanings, i.e., bank as a place to store and retrieve money or as the embankment of a river. Shifting flexibility is presumed to allow individuals to switch from one activated meaning of a homonym to another (Simpson and Kang, 1994; Gorfein et al., 2000) when required by the task (see **Figure 2** for a sketch of the resulting task).

**Figure 1:**
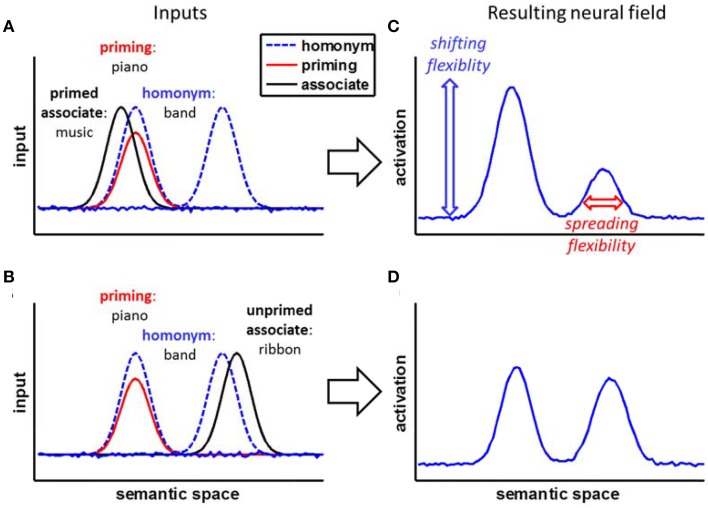
**A conceptual neural field framework for the homonym relatedness judgment task. (A,B)** The homonym input activates two meanings: one meaning is primed by previous information. The associate is either placed in the primed semantic space **(A)** or the unprimed semantic space **(B)**. **(C,D)** Within the neural field, inputs add up, leading to an initial advantage of the primed information. Shifting flexibility (a lower stability and amplitude of the priming) would allow letting loose of this initial priming and switch between the homonym's meanings, reducing effects of initial priming, while spreading flexibility would allow efficient searching for weakly related meanings, reducing effects of strength of association.

To illustrate our reasoning about the processes involved, Figure 1 sketches a simplified neural framework that builds on previous competitive activation models of ambiguity resolution (e.g., Plaut and Booth, 2000; Twilley and Dixon, 2000; Rodd et al., 2004). The framework assumes word meanings to be represented by patterns of activation in a dynamic neural field (Amari, 1977; Erlhagen and Schöner, 2002; Erlhagen and Bicho, 2006). The neural field represents the meaning of words distributed (Rodd et al., 2004) along a topographic semantic space (Plaut and Booth, 2000) with related word meanings positioned near to each other.

For homonyms, the two meanings are presented in separate subspaces of the field, assuming that a homonym's meanings are distinct. For example, the homonym *band* would have two separate peaks representing the meaning of a *small orchestra* and the meaning of a *tie*. Activation within the field is not limited to single points, but spreads from one unit to neighboring units—representations of concepts are, hence, diffuse and always co-activate related concepts that lie nearby in semantic space (compare Quillian, 1967). The more related two concepts are, the nearer they are to each other in the field and the stronger they co-activate each other. For example, the words *band* and *music* are nearer to each other and, hence, spread more activation to each other when activated, than the words *band* and *chord*. The priming of one of the two meanings of the homonym results in stronger initial activation of the primed semantic subspace of the whole homonym meaning space. For example, when the homonym *band* is shown and before, the context of music was primed, the peak representing the *small orchestra* would be supported by a decaying peak representing this priming.

Within this simplified neural framework with spreading activation within the field and the decaying effect of priming, we can derive two predictions for how fast a related word (the *associate* in the following) could be identified by the model. First, the nearer the associate is located to the homonym's core meaning, the better its identification is supported by the spreading activation from the homonym. For example, the relation between *band* and *music* is identified more quickly than the relation between *band* and *chord*. This difference represents the *spreading flexibility*: broader spread of activation should support the identification of words less related to the core concept activated by the homonym.

Second, an associate from within the primed subspace can be identified more easily than an associate from within the unprimed subspace, although the influence of priming is expected to decay over time. For example, the word *music* would be identified more rapidly than the word *ribbon*. This difference represents the shifting flexibility: The more stable the neural representation of the primed homonym meaning is, the more difficult it is to shift to a completely different meaning (this closely matches the implementation of shielding in common neural network models, e.g., O'Reilly, 2006; Herd et al., 2014).

To tackle these two types of cognitive flexibility empirically, we created a task that asked participants to identify associates that have first either been primed or unprimed, and second are either strongly or weakly related to the homonym. From the theory as sketched in the neural framework, we can derive the following empirical hypotheses (for a formal implementation and simulation of the framework in a dynamic neural field model and the resulting predictions, see Supplementary Material Data Sheet 1). First, we expect, that there will be independent main effects in response times and error rates of priming and association indicating the independence of the two types of cognitive flexibility. Second, we expect that the two types will influence participants' mouse movements at different points in time: the priming should be influential at an earlier time than the strength of association and both should not interact while active in parallel. This difference in timing and the temporal independence would provide evidence for the independence of the underlying processes. Third, on an inter-individual level, we expect no correlation across participants between the two types. Notably, due to the deliberately chosen sample size, this can only be seen as weak and additional, but not crucial evidence.

## Method

### Participants

Twenty students (10 female, mean age = 24.7 years) of the Technische Universität Dresden took part in the experiment. All participants had normal or corrected to normal vision. They received class credit or 5 € payment. The study was performed in accordance with the guidelines of the Declaration of Helsinki and of the German Psychological Society. An ethical approval was not required since the study did not involve any risk or discomfort for the participants. All participants were informed about the purpose and the procedure of the study and gave written informed consent prior to the experiment. All data were analyzed anonymously.

### Materials, apparatus, and stimuli

The presented material consisted of 252 related/associated and 252 unrelated word-pairs (for some of these pairs existed several versions due to different conditions, see below).

**Figure 2:**
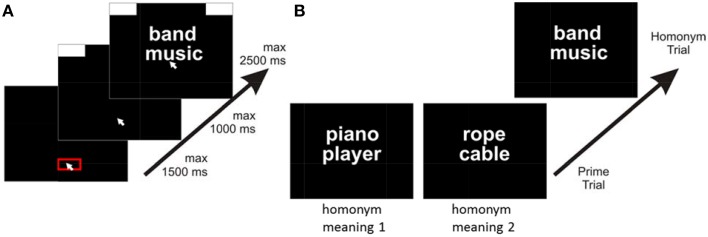
**Setup of the experiment**. **(A)** Sequence within trials. Participants started a trial by clicking into a red box at the bottom of the screen. After clicking, response boxes appeared at the upper edges of the screen and participants had to move the cursor upwards, in order to start presentation of stimuli. After reaching a movement threshold, two words appeared (here the homonym and the associate) and participants had to move the mouse cursor to the left or the right response box to indicating relatedness or not. **(B)** Sequence across experimental trials. Prime trials presented associated words that activated one or the other meaning of a homonym presented in the following homonym trial. Words in the homonym trial could be either strongly associated or weakly associated (here: strong association).

The trials of interest were so called homonym trials (84 in number) that consisted of a German homonym (target word) paired with a related word (the associate), which existed in four versions derived from two factors: the associate was either *strongly associated* with the respective homonym meaning or it was *weakly associated*; and the associate was either related to *one meaning* of the homonym or the *other meaning*. Each of the homonym trials was preceded by a prime trial (84 in number) consisting of two related words which existed in two versions: One of the prime trial versions was priming one meaning of the homonym; the other prime trial version was priming the other meaning of the homonym (see Figure 2B).

To avoid participants learning and adapting to this prime-homonym structure in the sequence of trials, we added filler trials using further material: associated filler trials (84 in number), with two associated words; homonym catch trials with a homonym and an unrelated word (84 in number); filler catch trials with two unrelated words (168 in number). The order of the different trial types was random with the exception that prime trials and homonym trials always followed each other. The experimental material (prime and homonym trials) was evaluated in earlier studies (compare Dshemuchadse, 2009) and can be found in the Supplementary Material Table 1.

Additionally, we had 10 independent raters evaluate all of the material (including the filler and catch trials) for relatedness. All words presented together as target and associate in the different conditions were rated for relatedness on a scale from 1 (“no relatedness”) to 9 (“very strong relatedness”). The order of presentation was randomized across raters. Table 1 shows the average results across word pairs. The data indicate the intended strong relatedness for priming trials, strong homonym trials and filler trials (6.19–6.92), medium relatedness for weak homonym trials (4.43–4.77) and almost no relatedness for filler catch and homonym catch trials (1.04–1.17). Furthermore, we calculated for each pair of strong associates (trial set A and B) the difference between their homonym relatedness to estimate the degree to which the homonym meanings were balanced. For the average difference, the low absolute value of 0.15 indicates well balanced homonym meanings across the two homonym trial sets A and B. For the average of the absolute difference, the relatively low value of 1.16 indicates a sufficient general balance of the homonym meanings (for the complete specific word pair ratings, see the Supplementary Material Table 1).

**Table 1:** **Relatedness Ratings (1 = unrelated, 9 = strongly related) for the experimental homonym trials and the other four types of trials (priming, homonym catch, filler, and filler catch) for both sets of homonym meanings (A and B)**.

	**Homonym Trials**					
	**A strong**	**A weak**	**B strong**	**B weak**	**A-B strong**	**ABS (A-B) strong**
Mean	6,77	4,43	6,92	4,77	−0,15	1,35
SD	1,33	1,83	1,08	1,77	1,78	1,17
	**Priming trials**		**Homonym**		**Filler**	
	**Set A**	**Set B**	**Catch**	**Assoc**.	**Catch**	
Mean	6,25	6,57	1,04	6,19	1,17	1,16
SD	1,66	1,30	0,90	1,45	0,97	1,12

*For homonym trials, the average difference of strong associates indicates balancing of the two homonym meanings across the two sets of meaning A/B; the average absolute difference indicates the degree of general balance between the two homonym meanings*.

Stimuli (target word and associate) were presented in white on a black background on a 17 inch screen running at a resolution of 1280 × 1024 pixels (75 Hz refresh frequency). Words were printed in Arial (font size: 48 pt) in the horizontal center of the screen. The target word was present 100 px above the vertical center, the associate 35 px below the vertical center (this upwards bias was chosen to suit the upwards direction of mouse movements). Response boxes (200 px in width) were presented at the top left and top right of the screen. For presentation, we used Psychophysics Toolbox 3 (Brainard, 1997; Pelli, 1997), Matlab 2006b (the Mathworks Inc.), and Windows XP. Responses were carried out by moving a computer mouse (Logitech Wheel Mouse USB), sampled with a frequency of 92 Hz.

### Procedure

Participants' task on each trial was to judge whether the two words presented were related or not. After onscreen instructions and demonstration by the experimenter, participants practiced 20 trials, followed by the main experiment (material of these practice trials did not appear again in the experiment).

Each trial consisted of three stages (see Figure 2A), following an established mouse task procedure (Scherbaum et al., 2010; Dshemuchadse et al., 2012). In the first stage, participants had to click into a red box (140 px in width) at the bottom of the screen within a deadline of 1.5 s. This served to produce a comparable starting area for each trial. After clicking within this box, the second stage started and two response boxes at the right and left upper corner of the screen were presented. Participants were required to start the mouse movement upwards within a deadline of 1 s. We chose this procedure forcing participants to be already moving when entering the decision process to assure that they had not already decided prior to simply executing the final movement. Only after participants moved the cursor at least 4 pixels in each of two consecutive time steps the third stage started with the appearance of the target word (e.g., the homonym) and the associate (hence, the time for stages 1 and 2 could be conceptualized as the inter-trial-interval). Participants were instructed to move the cursor into the upper left response box to indicate that both words were related and into the upper right box to indicate that both words were not related (directions were balanced across participants).

The trial ended after moving the cursor into one of the response boxes within a deadline of 2.5 s (see Figure 2). If participants missed the deadline of one of the three stages, the next trial started with the presentation of the red start box. Response times (RT) were measured as the duration of the third stage, reflecting the interval between the onset of the target stimulus and reaching the response box with the mouse cursor.

### Design

The experiment consisted of 5 types of trials (prime, homonym, filler, catch homonym, and catch filler—see Materials, Apparatus, and Stimuli). The main experimental manipulation concerned the consecutive prime and homonym trials. Prime trials primed one of the two meanings of the homonym in the following homonym trials. Homonym trials consisted of the homonym and an associate that was either strongly associated or weakly associated with either one or the other of the homonym's meanings. Across two blocks of trials, participants experienced each homonym twice, with each meaning primed once (order of primed meanings was balanced across participants). Priming and strength of association were manipulated orthogonally (randomized), leading to a 2 (primed/unprimed) × 2 (strong/weak) design with 42 trials per condition. As a control factor, we included *repetition*, the first and second presentation of the homonym in the analysis.

### Data preprocessing

For analysis of RT and mouse movements, we excluded erroneous trials (compare **Figure 7**) and trials aborted because of participants clicking too late into the start box (0.14%) or starting their movement too late (0.01%). On average, the inter-trial-interval (stages 1 and 2 of the mouse procedure) lasted 1068 ms (*SE* = 41 ms).

We aligned all movements for a common starting position (within the range of the start box) and normalized each movement to 100 equal time slices (Spivey et al., 2005; Scherbaum et al., 2010). For a detailed analysis of the time course of influence on mouse movements, we used the mouse movement trajectory angle on the XY plane (compare Scherbaum et al., 2010) as dependent variable. We calculated the angle relative to the y-axis for each difference vector between two time steps^1^. Following this, we prepared the temporal analysis by introducing temporal correlation between the single data points. To this end, we filtered the data with an 8-point Gaussian smoothing window across time steps (the 8-point window was chosen to equal the correction criterion for multiple testing as explained in the Results section).

## Results

### Discrete results for homonym trials

**Figure 3:**
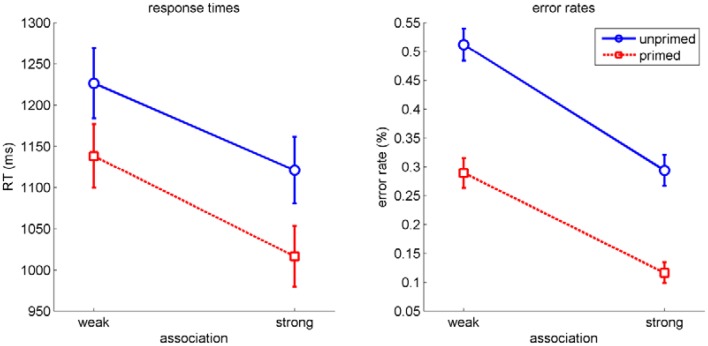
**RT (left) and error rates (right) in homonym trials as a function of association and priming**. Errorbars indicate standard errors.

Our main experimental interest was in the dynamics of the two influences within homonym trials. However, as a first check for successful manipulation and for independence of the two factors *priming* and *association*, we performed a repeated measures analysis of variance (ANOVA) on RT and on error rates with the factors *priming* (primed/unprimed), *association* (strong/weak), *repetition* (first experience/second experience). For RT (see Figure 3, left), this revealed significant main effects for *priming, F*_(1, 19)_ = 129.93, *p* < 0.001, η^2^_*p*_ = 0.87, *association, F*_(1, 19)_ = 84.28*, p* < 0.001, η^2^_*p*_ = 0.82, but no effect for *repetition, F*_(1, 19)_ = 0.02, *p* = 0.9. There was no significant interaction (all *p*>0.1). For error rates (see Figure 3, right), the analysis yielded comparable results (*priming* and *association: p* < 0.001, all other *p*>0.09). Hence, priming and association influenced processing independently of each other. Furthermore, the insignificant control factor indicates that the second experience of the word material did not influence participants' processing substantially.

Since, the error rates were at the 50% chance level in the condition *unprimed-weak*, we aimed to ensure that participants performed the task correctly and did not simply respond randomly. Therefore, we performed a signal detection analysis (Green and Swets, 1966) with these most difficult homonym trials and the homonym catch trials: We coded relatedness as signal and participants' choices as decision. If participants decided randomly in difficult trials, we would expect them to show sensitivity near to zero. However, the analysis revealed a mean sensitivity of 1.41 (*SE* = 0.11, *t* = 12.95, *p* < 0.001). We interpret this as evidence that participants performed the task correctly, even when they erred often in the most difficult condition.

**Figure 4:**
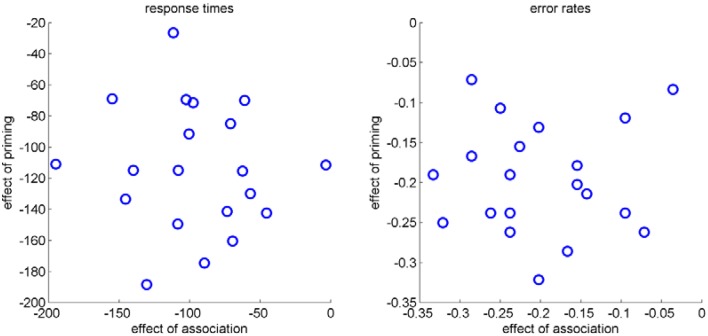
**Scatter plots of the effect of association (strong-weak) and the effect of priming (primed-unprimed) for RT (left) and error rates (right) of each participant**.

With this basic pattern as expected, corroborating the independence of the factors priming and association would usually mean looking at inter-individual variability and to calculate correlations for these factors across participants. Although we measured only 20 participants, we performed this analysis for RT and error rates (see Figure 4). Results for RT, *r* = −0.08, *p* = 0.75, and error rates, *r* = 0.04, *p* = 0.88, indicated no correlation between the two effects across participants. As noted in the introduction, this correlational approach usually relies on larger number of participants. The analysis of within trial dynamics as indicated by mouse movements circumvents these disadvantages, as described in the following sections.

### Continuous results for homonym trials

**Figure 5:**
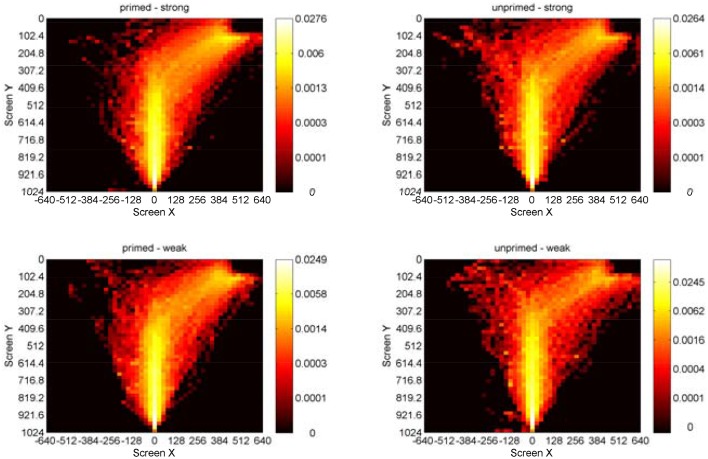
**Heatmaps showing log10 probabilities of correct-response mouse movements in homonym trials on the XY plane for the factors priming and association (pooled for all participants)**. Participants started at the bottom center and moved to the upper-right response box.

As a first validating analysis, we analyzed mouse movements for the same effects as found in RT and errors (see Figure 5). To this end, we calculated the degree of curvature calculated as the area under the curve between a direct straight line movement and the real curved movement in a trial. An ANOVA on the degree of curvature for the factors *priming* (primed/unprimed), *association* (strong/weak), *repetition* (first experience/second experience) yielded significant main effects for *priming, F*_(1, 19)_ = 100.96, *p* < 0.001, η^2^_*p*_ = 0.84, *association, F*_(1, 19)_ = 60.01*, p* < 0.001, η^2^_*p*_ = 0.76, but no effect for *repetition, F*_(1, 19)_ = 0.27, *p* = 0.61. There was no significant interaction (all *p*>0.1).

**Figure 6:**
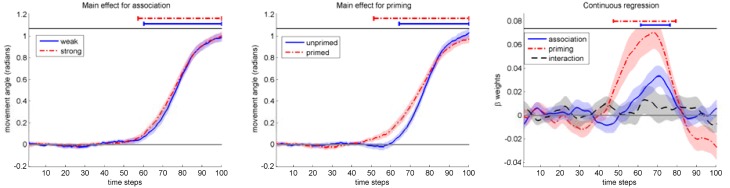
**Left and middle: Mouse movement angle for the conditions association (left) and priming (middle)**. **Right:** Results of continuous regression on mouse movement angle. Bars above data indicate significant *t*-tests against zeros. Shaded areas indicate standard-errors.

We then performed the main analysis for mouse movements in homonym trials by time continuous multiple regression (Notebaert and Verguts, 2007; Scherbaum et al., 2010) on mouse movement angles (see Figure 6, left and middle panel; additionally, see the Figure in the Supplementary Material Image 1 for the other types of trials) with three regressors: *association* (strong/weak), *priming* (primed/unprimed) and the interaction *association* × *priming*. The first two regressors were normalized to a range of [−1, 1]. To exclude multicolinearity as a source of artifacts, we checked *variance inflation factors* to stay below 1.1, indicating the necessary low level of multicolinearity.

We calculated 100 multiple regression analyses (100 time slices → 100 multiple regressions) yielding three time-dependent beta weights (three weights across 100 time slices) for each participant. For each of these three beta-weights, we computed grand averages representing the time-varying strength of influence for each predictor (see Figure 6, right). To analyze the properties of these three beta-weights, we checked for relevant temporal segments of influence by calculating *t*-tests against zero for each time step of these beta-weights (Scherbaum et al., 2010; Dshemuchadse et al., 2012). To compensate for multiple comparisons of temporally dependent data, we followed previous studies (Scherbaum et al., 2010; Dshemuchadse et al., 2012) and chose as a criterion of reliability a minimum of eight consecutive significant *t*-tests (see Dale et al., 2007 for Monte Carlo analyses on this issue).

The results (see Figure 6, right, and Table 2) indicate that *association* and *priming* followed different time courses. The influence of priming started earlier than association [*M*(RT) = 552 ms vs. *M*(RT) = 716 ms], as we expected. The *interaction* between both factors did not show any significant temporal segments of influence.

**Table 2:** **Timing of the influence of priming and association on mouse movement angles**.

**Significant temporal segments**		
**REGRESSOR**		
Priming	Time steps	47–79
	M(RT)	552–927 ms
Association	Time steps	61–76
	M(RT)	716–892 ms
Priming × Association	Time steps	–
	M(RT)	–

### Results across all types of trials

**Figure 7:**
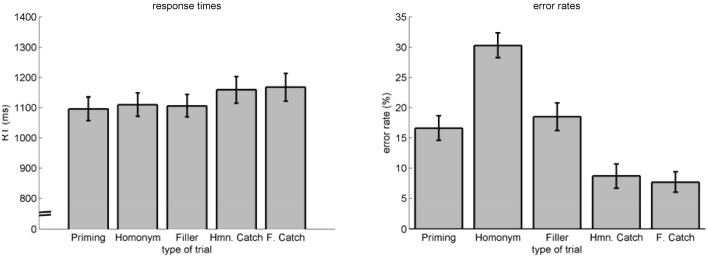
**RT (left) and error rates (right) as a function of trial type**. Errorbars indicate standard-errors.

Our main focus was on the experimental investigation of the dynamics within the homonym trials. To check for the validity of the overall experiment, we also analyzed RT and error rates of the different types of trials (see Figure 7).

We performed an ANOVA on RT for the factor *trial type* (priming, homonym, filler, homonym catch, filler catch), revealing significant differences *F*(1.62, 30.75)^2^ = 14.83, *p* < 0.001, η^2^_*p*_ = 0.44. *Post-hoc t*-tests revealed this difference to be located between *priming, homonym*, and *filler* trials on the one side and *homonym catch* and *filler catch* trials on the other side (all *p* < 0.01, uncorrected). Hence, this effect indicates that finding an association was easier than rejecting any association. Concerning error rates, an ANOVA also revealed significant differences for the factor *trial type F*(1.42, 26.94)^2^ = 33.57, *p* < 0.001, η^2^_*p*_ = 0.64. *Post-hoc t*-tests revealed *priming* (*M* = 16.64%, *SE* = 2.03%) and *filler* (*M* = 18.51%, *SE* = 2.28%) trials to be similar, as well as *homonym catch* (*M* = 8.72%, *SE* = 2%) and *filler catch* (*M* = 7.72%, *SE* = 1.68%) trials. *Homonym* (*M* = 30.03%, *SE* = 2.05%) trials were significantly different to all other trials (all *p* < 0.001). *Homonym catch* and *filler catch* were different to all non-catch trials (all *p* < 0.01). Again, finding an association was different to rejecting any association—the lower error rate for catch trials indicates, however, a speed-accuracy trade-off: instead of risking missing an association, participants seemed to double check before responding that no association was present.

## Discussion

The aim of our study was to decompose cognitive flexibility as a component of executive functions into two distinguishable types, namely shifting flexibility—the readiness to switch between cognitive sets—and spreading flexibility—the ability to identify related cognitive sets. To overcome the limitations of inter-individual correlational approaches, we chose an experimental approach using a homonym relatedness judgment task combined with mouse movements. The former served to manipulate the two types within the same task; the latter allowed us to dissociate the two types by their temporal variance within trials.

Our results indicate that the manipulation of the two types leads to independent effects. This independence is reflected on the one hand in distinct temporal patterns of influence within trials as measured with mouse movements, and on the other hand in independent correlational patterns of RT and error rates across participants. These results match the predictions theoretically based on a neural framework assuming continuous representations of word meanings in a neural field (for a formal implementation and simulation of the predictions, see Supplementary Material Data Sheet 1).

Our use of filler trials, additional to the central homonym trials, indicated several additional findings that validate the findings presented here. First, homonym trials showed a higher error rate than all other trials, indicating that working against the priming of the wrong context and identifying weakly related associates were a difficult task for our participants compared to the standard association trials. Second, in catch trials, when no relatedness between the two words was present, participants were slower but showed less errors. This finding supports the assumption that participants aimed at identifying associations (instead of the opposite strategy of excluding associations) and only responded with judgments of no relatedness after checking twice. This also indicates, that despite the high error rates, participants did not simply guess in the most difficult trials (unprimed—weakly related associates), but still performed the task they should have performed so that mouse movements in correct trials still contain the trace of the processes of interest.

It has to be noted that our participants experienced all homonyms twice to increase the absolute number of trials. In light of previous findings, i.e., of primacy effects in ambiguity resolution (Gorfein et al., 2000), it was important that our analyses indicated no substantial difference between the first and the second experience.

Could our results also be explained by priming effects as they would also be present in a simple word/ non-word recognition task? While this would question our interpretation of the priming effect as an indicator of shifting-flexibility, results from pretests (Dshemuchadse, 2009) contradict such an interpretation. When we varied the temporal presentation order of the associate and the homonym, priming effects were much larger when the homonym was presented first (as in the study reported here). Hence, processing the homonym built up an expectation that participants had to overcome to identify the unprimed associated and this exactly matches our definition of shifting flexibility.

Beside the central findings of our study, three further theoretical and methodological implications for psychological research can be discussed.

First, our results and the underlying theoretical framework question the reduction of cognitive flexibility to one single construct, namely shifting flexibility. Representing cognitive flexibility with only one parameter, i.e., the neural gain parameter in both task switching (O'Reilly, 2006; Herd et al., 2014) and ambiguity resolution (Plaut and Booth, 2000) confounds the two subtypes: In neural field models, manipulating the gain parameter leads to changes in both, stability and breadth (specificity) of activation and hence to a dependence of shifting and spreading flexibility as defined here. In the light of our results, this dependence can be questioned. In our framework, we assumed shifting flexibility to be related to the stability of neural activation patterns as reflected in the strength of neural self-excitation. In contrast, spreading flexibility was related to the breadth of the spread of neural activation. Concerning the conceptualization of control dilemmas, more complex elaborations (Goschke, 2012) indeed distinguish between a shielding-shifting and a selection-monitoring dilemma, pointing to different underlying parameters. Our results support this distinction and show that although the common effects of positive mood might indicate only one dilemma (Goschke and Bolte, 2014), the distinction should be made. With our paradigm, we show that it is possible to meet both demands: to shift cognitive sets and to spread across cognitive sets. Furthermore, we suggest that the selection-monitoring dilemma can be extended from the breadth of attention to breadth of activation in the semantic space (compare Rowe et al., 2007). Notably, the complementary nature of the dilemmas implies that any benefits of a certain configuration—for both, shifting and spreading flexibility—also come with costs: In accordance with this, we assume for shifting flexibility that a smaller effect of priming implies not only easier switching but also less benefit when staying; concerning spreading flexibility, we expect that a broader spreading could lead to difficulties when focusing on one concept or when distinguishing concepts is necessary.

Second, the way we implemented shifting and spreading flexibility suggests that the difference between these two types in our experiment cannot be reduced to other distinctions of cognitive flexibility or cognitive control. Concerning the latter, a distinction between proactive and reactive control had been propose previously (Brown et al., 2007; Braver, 2012). However, whether subjects had to switch to the unprimed meaning of the homonym (shifting flexibility) or whether they had to search for the weak association (spreading flexibility) was only evident when the word-pairs appeared on the screen. Hence, they were unforeseeable and in both cases triggered externally or under reactive control. In terms of cognitive control processes the proposed difference between shifting and spreading flexibility could be mapped to a distinction between a switching component and a searching component. This latter distinction is related to two types of cognitive flexibility as proposed by Eslinger and Grattan, namely reactive flexibility and spontaneous flexibility (Eslinger and Grattan, 1993). However, these types are assumed to be more general than the types of flexibility, we propose here. Especially, while reactive flexibility refers to the instructed or demanded shifting of cognitive sets as we specify for the *shifting flexibility*, spontaneous flexibility refers to the free search for knowledge bypassing automatic responses in order to attend to more divergent ideas, thus going beyond our definition of *spreading flexibility*. This difference could be due to the different methods: while Eslinger and Grattan's types of flexibility are based on the study of brain lesions, we used an experimental approach that was based on neural parameters. It is an open question if a combination of the different types of cognitive flexibility might lead to a finer differentiation matching both definitions.

Third, we provided a methodological alternative to the correlational approach common in research on executive functions by combining within-task manipulations with mouse movement analyses. Instead of correlations across participants, the analysis of variance and temporal dynamics in trials allowed us to examine the distinction of shifting and spreading flexibility with a small sample of participants.

Overall, we argue that distinguishing two types of cognitive flexibility, namely shifting and spreading flexibility, as components of executive functions could reveal new insight into the process underlying flexible, goal oriented behavior. Furthermore, we present a continuous relatedness judgment task facilitating further research in the intersecting field of executive functions and semantic processing.

## Conflict of interest statement

The authors declare that the research was conducted in the absence of any commercial or financial relationships that could be construed as a potential conflict of interest.

## Footnotes

^1^Mouse movement angle has two advantages over the raw trajectory data as a measure of movement tendency. First, it better reflects the instantaneous tendency of the mouse movement since it is a differential measure compared to the cumulative measure represented by raw trajectory data. Second, it integrates the movement on the XY plane into a single measure.

^2^Greenhouse-Geisser corrected.

## Supplementary material

Image 1 **Mouse movement angle for the interaction of the two experimental conditions association and priming in the homonym trials and the two additional conditions filler trial (not associated) and prime trial (associated)**. Shaded areas indicate standard-errors.
