# Supplementary figures and images for: Action dynamics reveal two types of cognitive flexibility in a homonym relatedness judgment task
Source: Front Psychol. 2015 Aug 28;6:1244. doi: 10.3389/fpsyg.2015.01244 (PMC4551828; doi:10.3389/fpsyg.2015.01244)

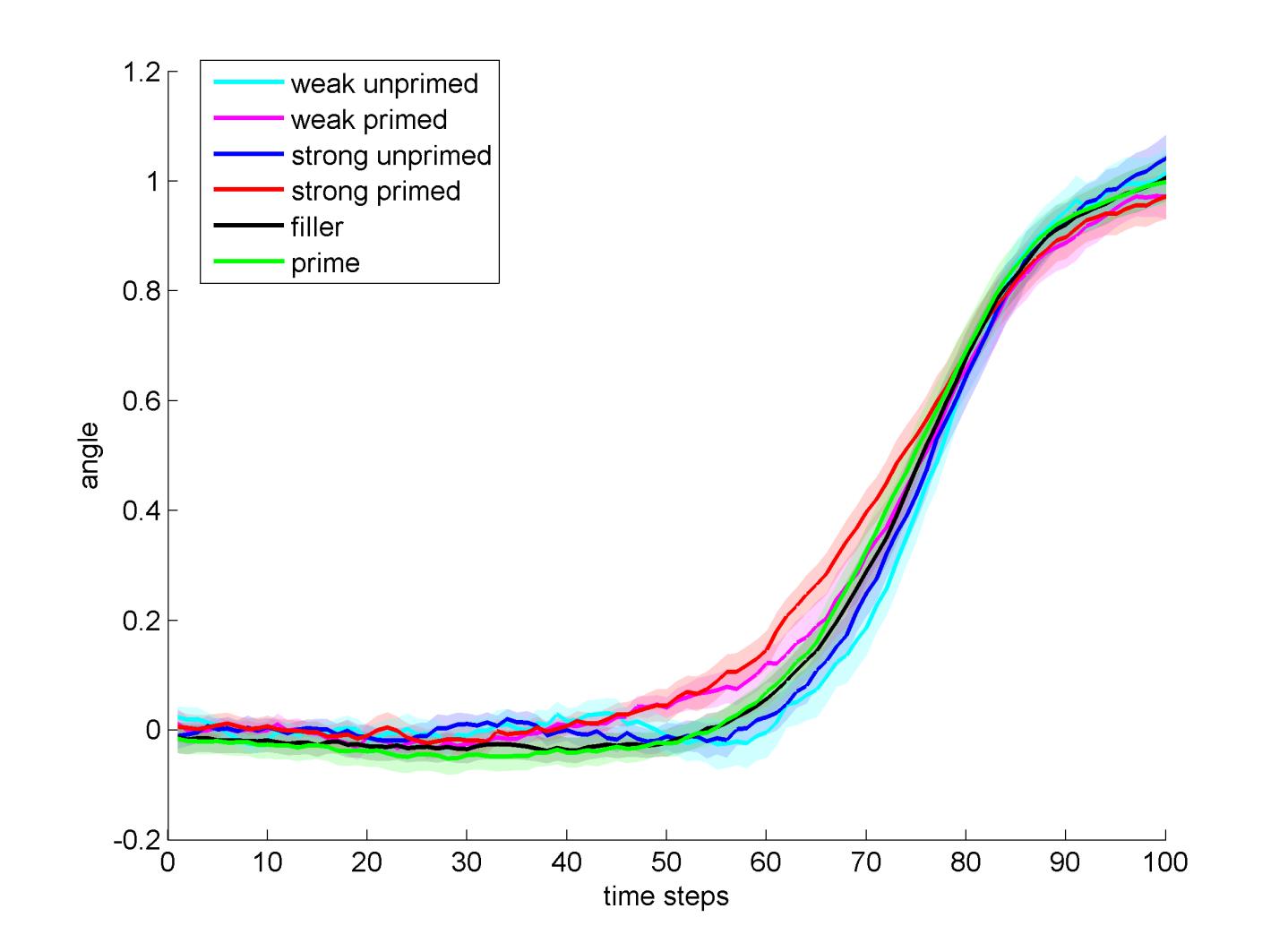

Supplement: Image 1 — Mouse movement angle for the interaction of the two experimental conditions association and priming in the homonym trials and the two additional conditions filler trial (not associated) and prime trial (associated). Shaded areas indicate standard-errors. [file Image1.JPEG]
