# Supplementary material for: Action dynamics reveal two types of cognitive flexibility in a homonym relatedness judgment task
Source: Front Psychol. 2015 Aug 28;6:1244. doi: 10.3389/fpsyg.2015.01244 (PMC4551828; doi:10.3389/fpsyg.2015.01244)
Supplement: Supplementary file 3 [file DataSheet1.DOCX]

# Appendix: A simple neural field model of the homonym relatedness judgement task

To derive predictions based on the theory as outlined in the introduction, we implemented a simplified neural field model of the proposed processes underlying reactive and spontaneous flexibility in the homonym relatedness judgement task. The simulation based on this model could be seen as a run in the experiment by an ideal participant.

## Structure of the model

The model consisted of one neural field layer. Activation *u* of units *x* at time *t* in the layer followed the self-stabilizing linear differential equation

|  | (1) |
| --- | --- |

with the time scale *τ* = 10, the resting level *h* = -5, the input *s*, and spatially correlated Gaussian noise *η*.

Inputs *s* were defined by setting the respective locations *x* to 1 and convolving the resulting impulses with a Gaussian interaction kernel *w*

|  | (2) |
| --- | --- |

With *w_e_* = 3 representing the excitatory strength of the input and *σ* = 5 defining the breadth of the interaction kernel. Similarly, the level of noise was also set to *η* = 3. Each influence, the homonym, the priming of a homonym meaning, and the associate, were independent inputs of the strength *w_e_.*

## Simulation

We simulated homonym-trials in a typical experimental setup with the four conditions (*association x priming*) strong-primed, strong-unprimed, weak-primed, weak-unprimed as indicated in Figure 1. Simulations were performed in Matlab 2010b (the Mathworks, Inc.).

### Stimuli

We used a layer with 100 units. Locations for the inputs of the homonym (see Figure A1) within this layer were set to *x_homonym1_* = 35 and *x_homonym2_* = 65, for the residual priming to *x_priming_* = 35, and for the different combinations of primed/unprimed and weakly/strongly related associates to *x_associated_primed_strong_* = 35, *x_associated_primed_weak_* = 30, *x_associated_unprimed_strong_* = 65, and *x_associated_unprimed_weak_* = 70. In each simulated trial, only one of the associate inputs was present.


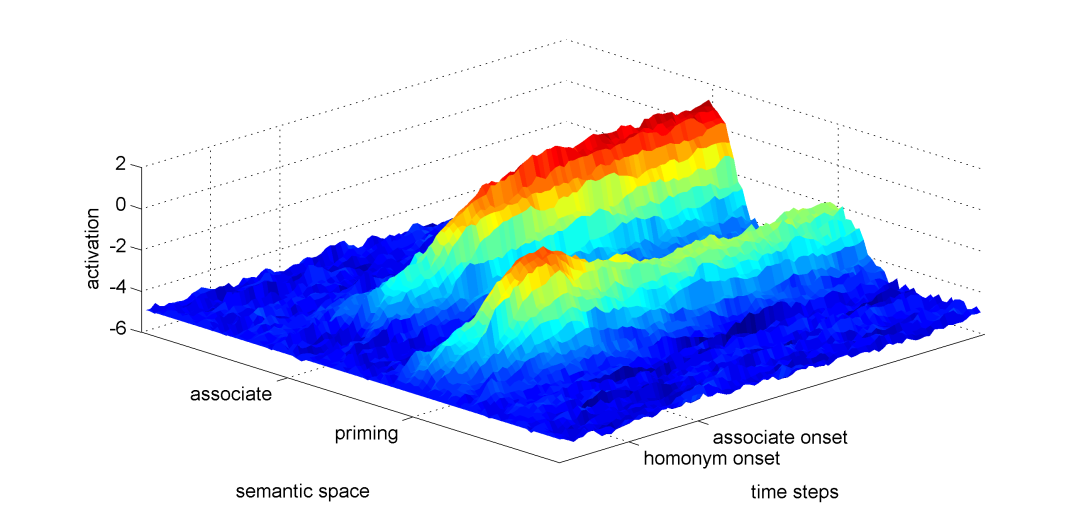


*Figure A1. Structure of the simplified neural field model in a simulated trial. Inactive neural units stay at a resting level, while activated units raise their activation above this resting level. In semantic space, a certain meaning of the homonym was primed by initial activation in the respective semantic subspace. The homonym's two meanings were represented as activation in both semantic subspaces. The associate was presented in one of these subspaces, either the primed one or, as shown in the figure, the unprimed one. Within this subspace, the associate was placed at different distances to the homonym’s activations, either at the same location (strong association, as shown in the figure) or 5 units aside (weak association).*

### Procedure

Each trial began with the priming input present at *t* = 1. At *t* = 15, the homonym was presented, followed by the associate at *t* = 30. This way, we simulated the order of word reading. The priming input was switched off at *t* = 30, simulating the initial presence of the residual activation and the decay when all new information was present. A response was given by the simulated movement trajectories reaching a response threshold (see next section).

For each simulated participant, we performed 20 trials with each of the 4 types of associates, resulting in 80 simulated trials.

### Individual runs

We performed 20 runs of the model, simulating 20 individual participants. To simulate inter-individual variation in reactive and spontaneous flexibility, we varied *w_e_* for the priming input and *σ* of all inputs by adding uniformed random variation of [-0.3,0.3] to the base *w_e_* of 3 and the base *σ* of 5. Furthermore, we simulated inter-individual variation in overall RT by choosing the time scale of movement generation *τ_mov_* (see next section) randomly from a uniform distribution [5,20].

### Calculation of movement trajectories

To simulate mouse movements of the simulated participants, we used an attractor dynamics approach as it is well established in cognitive robotics (Erlhagen & Bicho, 2006). This way, we continuously read out the continuous movement tendency of the model on a one dimensional virtual movement dimension (see Figure A2).

The movement dimension corresponds to the dimensions of the model’s layer (here: 100 units) and the start position x_mov_ is set at the (neutral) middle of the dimension: x_mov_ = 50.

To update x_mov_ , we follow a two-step procedure for every time point in the simulation. This procedure constructs a dynamic system from the signal in the neural field. This dynamic system exhibits an attractor at the peak of activation, if present.

In the first step, the position x_peak_ of the attractor is calculated by multiplying a ramp function along the *x-*axis with the activation peaks in the field, according to the formula

|  | (3) |
| --- | --- |

with *sig(u)* defining the non-linear activation function of the form

|  | (4) |
| --- | --- |

(in principle, this sigmoid activation function defines the neural output of the field: α = 0; *β* = 5).

For a single peak of arbitrary width, *x_peak_* locates the center of this peak. In the case of multiple peaks, *x_peak_* locates the nearest location between these multiple peaks indicating the best compromise for an indecisive movement.

In the second step, the tendency of movement of the system is determined by constructing a one-dimensional dynamic system with one fix point at x_peak_. The attractor is established by a Gaussian potential function similar to the Gaussian interaction kernel, with its peak at x_peak_ and a sigma of ½ of the size of the neural field.

|  | (5) |
| --- | --- |

x_mov_ is then updated by the derivative

|  | (6) |
| --- | --- |

Hence, it follows the movement tendency according to the potential function of equation 6 with a time scale of *τ_mov_* = *τ*. When x_mov_ reaches predefined target areas on the movement plane (left response: 30 < *x* < 40; right response: 60 < *x* < 70), a response is elicited and the trial ends.


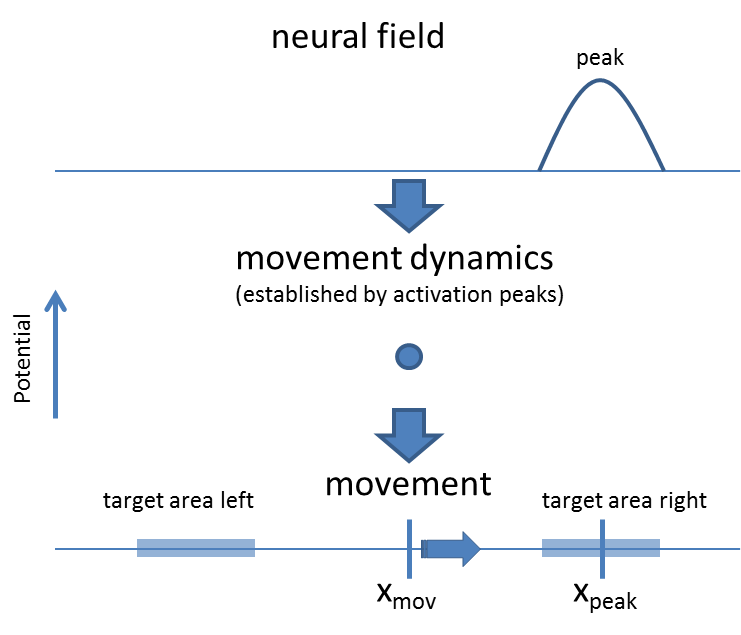


*Figure A2. Calculation of simulated movement trajectories. Activation peaks in the neural field yield a Gaussian attractor in a dynamic movement system at the position of the peak x_peak_. The current position on the movement dimension x_mov_ is updated by the change indicated by the dynamic system. When x_mov_ reaches a defined target area, the respective response is elicited.*

## Results

As shown in Figure A3, RTs of the model predict independent (additive) effects of priming (reactive flexibility) and association (spontaneous flexibility). This is also reflected in the continuous response tendency, as shown in Figure A4 (left and middle). Continuous regression of simulated response tendencies (see Figure A4, right) indicate an independent time course for both influence, with priming starting earlier in the trial and association.


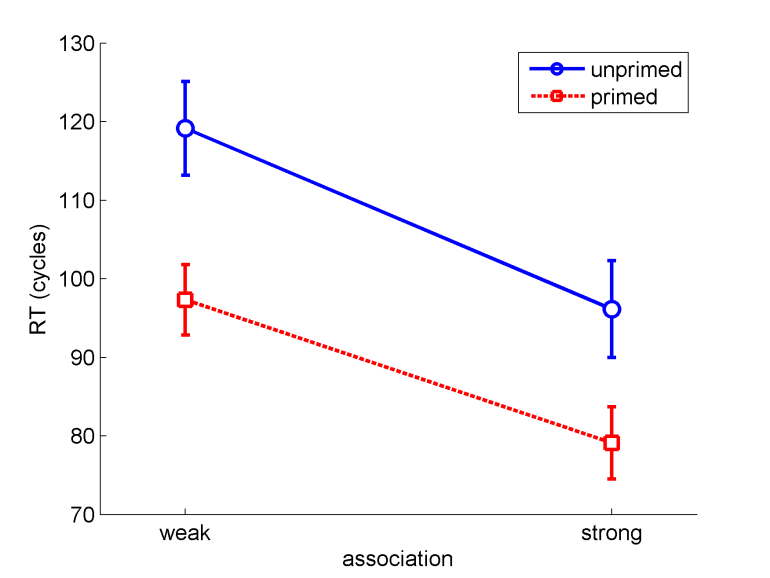


*Figure A3. RT in simulated homonym trials as a function of association and priming. Errorbars indicate standard errors across simulated participants.*


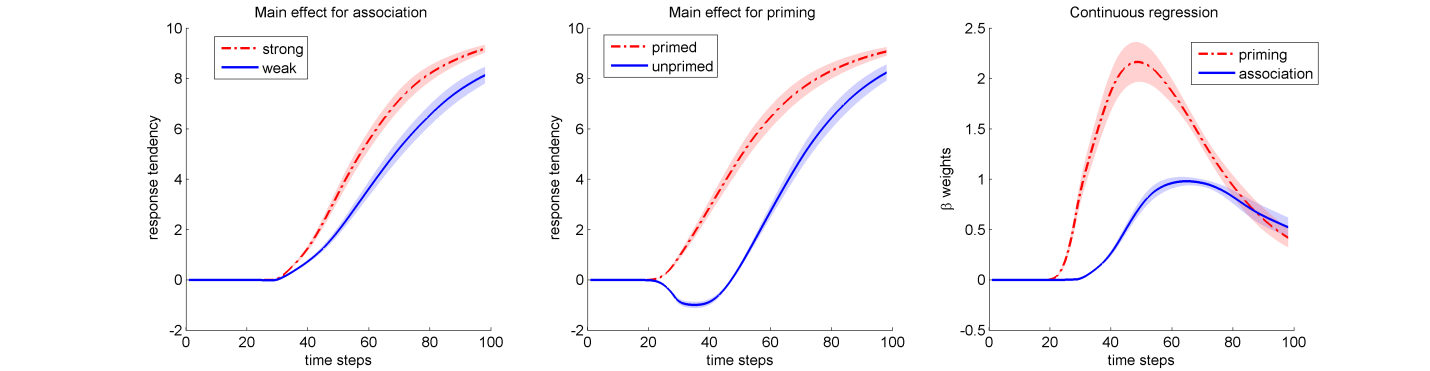


*Figure A4. Left and middle: Average movement tendency (normalized to mean RT cycles across participants) for the conditions association (left) and priming (middle). Right: Results of continuous regression on mouse movement angle. Shaded areas indicate standard-errors across simulated participants.*

Across simulated participants, there was no correlation of the main effects of priming (RT_unprimed_ - RT_primed_) and association (RT_weak_ – RT_strong_), *r* = 0.01 (see Figure A5), as could be expected since both parameters were varied independently.


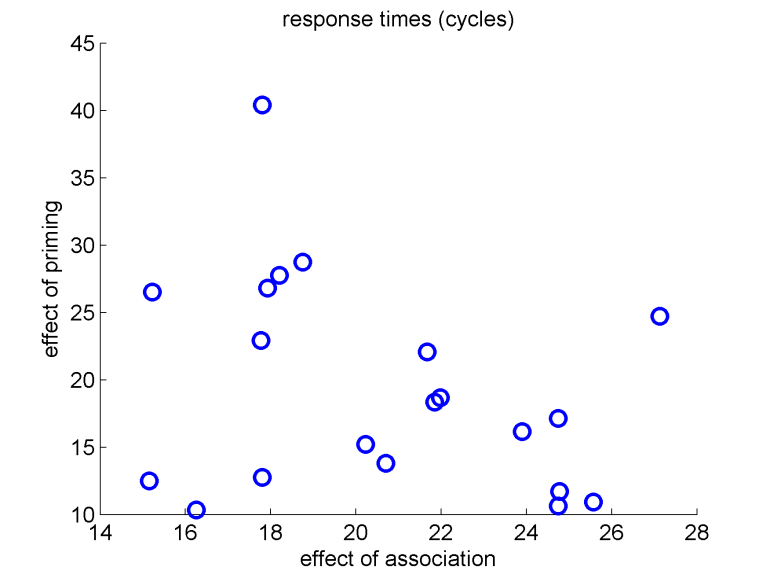


*Figure A5. Scatter plot of the association effects (strong-weak) and the priming effects (primed-unprimed).*

For a better comprehensibility of how RT and movement tendencies were produced by the model, Figure A6 shows the activation time course in the neural field of the standard model (without any inter-individual variation) for representative trials of each condition, overlaid by the corresponding response tendency (note that for analysis, primed responses were mirrored and all responses were set to a starting point of 0 so that the correct response was always positive).


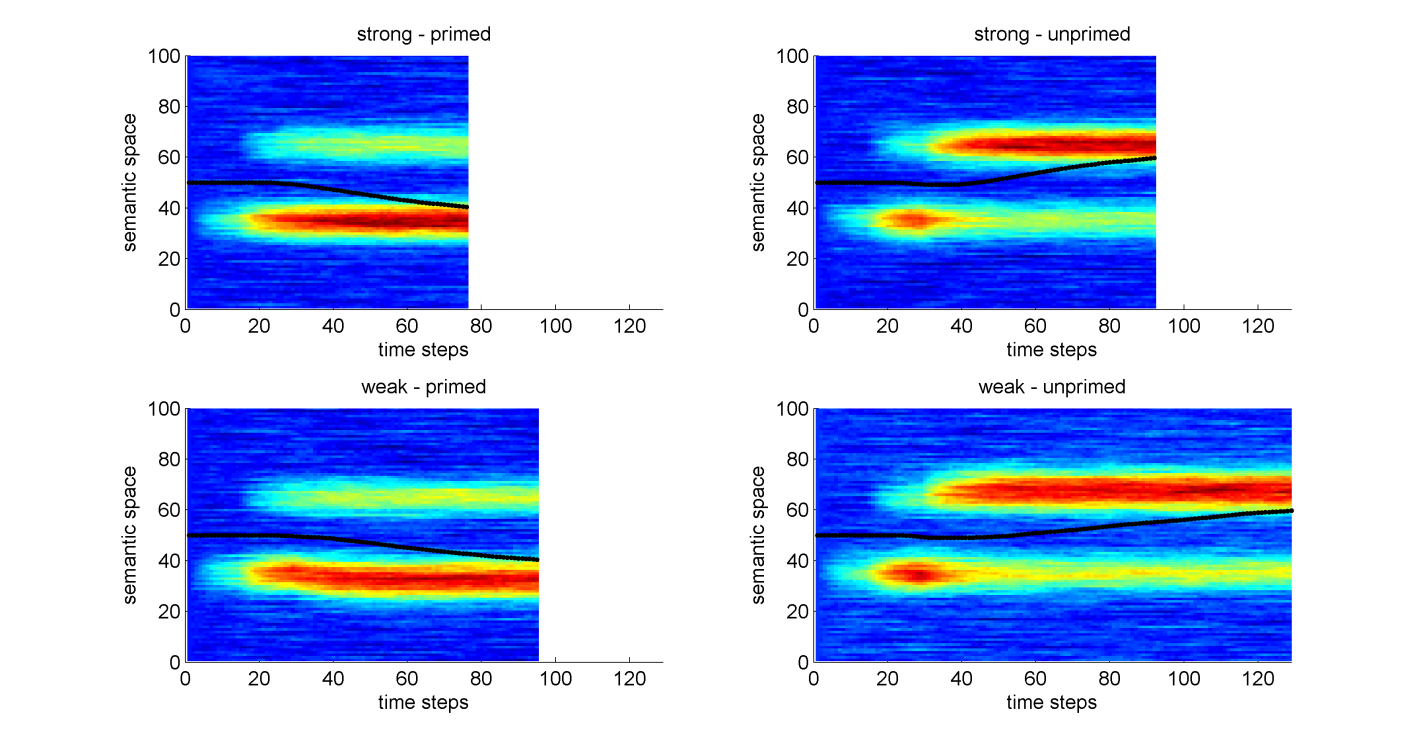


*Figure A6. Time course of neural activation for all four conditions of the base model (no random individual variation added). Primed meanings of the homonym are located in the lower semantic space. The black lined overlay indicated the continous response tendency of the model, with a neutral starting position in the middle of the field.*

**1.4 Summary**

The model predicts data patterns for two independent processes as proposed by our theory in the introduction. Notably, the model represents a simple extension of the activation Action dynamics and cognitive flexibility
